# Differential gene expression profiles in peripheral blood in Northeast Chinese Han people with acute myocardial infarction

**DOI:** 10.1590/1678-4685-GMB-2017-0075

**Published:** 2018

**Authors:** Lin Fan, Heyu Meng, Xudong Guo, Xiangdong Li, Fanbo Meng

**Affiliations:** 1 China-Japan Union Hospital, Jilin University, Jilin, China; 2 Medical College of Yanbian University, Yanji, China; 3 Department of Cardiovascular Medicine, China-Japan Union Hospital of Jilin University, Jilin, China

**Keywords:** acute myocardial infarction, RNA, differential expression

## Abstract

This study aimed to use gene chips to investigate differential gene expression profiles in the occurrence and development of acute myocardial infarction (AMI). The study included 12 AMI patients and 12 healthy individuals. Total mRNA of peripheral bloodwas extracted and reversed-transcribed to cDNA for microarray analysis. After establishing two pools with three subjects each (3 AMI patients and 3 healthy individuals), the remaining samples were used for RT-qPCR to confirm the microarray data. From the microarray results, seven genes were randomly selected for RT-qPCR. RT-qPCR results were analyzed by the 2^-ΔΔCt^ method. Microarray analysis showed that 228 genes were up- regulated and 271 were down-regulated (*p* ≤ 0.05, |logFC| > 1). Gene ontology showed that these genes belong to 128 cellular components, 521 biological processes, and 151 molecular functions. KEGG pathway analysis showed that these genes are involved in 107 gene pathways. RT-qPCR results for the seven genes showed expression levels consistent with those obtained by microarray. Thus, microarray data could be used to select the pathogenic genes for AMI. Investigating the abnormal expression of these differentially expressed genes might suggest efficient strategies for the prevention, diagnosis, and treatment of AMI.

## Introduction

Acute myocardial infarction (AMI) is one of the diseases with high mortality and morbidity globally. According to the World Health Organization (WHO), 17.5 million people died of cardiovascular diseases worldwide in 2012, accounting for 46% of the deaths caused by non-communicable diseases, and myocardial infarction was one of the major causes. The global incidence of AMI is rising owing to multiple reasons such as environment, heredity, or lifestyle. Various complications of AMI, such as cardiac rupture, arrhythmia, and ventricular aneurysm, have a great impact on patient life quality, and pose large economic burdens on the family and society.

Contemporary clinical epidemiology has shown that hypertension, diabetes, low density lipoprotein (LDL) lipoproteinemia, smoking, age, and gender are clearly associated with the occurrence of coronary heart disease (CHD), and so the accuracy of early CHD screening has been greatly improved. However, these traditional risk factors could not fully predict the onset and prognosis of AMI. The existing treatment methods such as anti-platelet aggregation therapy, lipid-lowering therapy, vasodilation therapy, or coronary stents have achieved great success in improving symptoms, delaying disease progression, and reducing mortality, but the desired therapeutic effects have not been obtained yet.

Inspired by the obvious characteristic of myocardial infarction (MI), namely familial aggregation, genetic studies on MI have made breakthrough progress in recent years. Studies have found that genetic effects could act as an independent factor influencing the onset of AMI (Qaseem *et al.*, 2012; Silbiger *et al.*, 2013; Tokat *et al.*, 2013; den Hoed *et al.*, 2015), and genetic polymorphisms are associated with the occurrence of MI (Karahan *et al.*, 2015; Salo *et al.*, 2015). Till date, 152 relevant sites and 320 candidate genes have been associated with increased risk of coronary artery disease (CAD) and AMI (The CARDIoGRAMplusC4D Consortium*et al.,* 2013; Do *et al.*, 2014; Welter *et al.*, 2014; Barth and Tomaselli, 2015). In studies on gene expression differences in myocardial cells of mice between AMI and sham groups *LOX, POSTN, SPARC, TIMP1*, and *SFRP2* were differentially expressed, and might have an impact on AMI (Wang *et al.*, 2015). Zhang Y*et al.* (2015) reported that *BNDF, PDGF-AA,* and *MMP-9* expression was up-regulated in ST-segment elevation myocardial infarction (STMI), and could therefore be used for the assessment of STMI. However, unlike traditional genetic disorders, MI is co-induced by a variety of genetic and environmental factors, as well as the interactions among them. So we have reasons to believe that a comprehensive gene-level analysis might be able to predict cardiovascular risks and make prognosis more accurately (Roberts and Stewart, 2012; Arvind *et al.*, 2015).

In the past decade, AMI onset has been identified to be associated with mutations in a number of genes involved in blood coagulation, the fibrinolytic system, platelet receptors, homocysteine metabolism, endothelial dysfunction, abnormal blood flow, and oxidative stress (Isordia-Salas *et al.*, 2010). A study concerning gene expression in myocardial cells of rats with AMI showed that immune response, chemotaxis, inflammation, cytoskeletal tissues, and other pathways were activated as early responses within 30 min of MI (Erdal *et al.*, 2012). However, most findings regarding myocardial cells are based on animal experiments, as human samples are difficult to obtain. It was previously reported that a polymorphism assay of peripheral blood-associated genes could predict the probability of CAD occurrence more accurately (Kathiresan *et al.*, 2008). Maciejak *et al.* (2015) compared the gene expression in peripheral blood of patients with AMI and non-MI CHD and found that some genes showed significantly different expression in the acute phase of MI, and the differences gradually disappeared with time. Therefore, in this study, we used peripheral blood as samples, which can be easily obtained and provide accurate results, to determine the genes that were differentially expressed in Han patients with AMI from northeastern China, so as to find new targets for the treatment of MI in Northeast Chinese Han patients by verifying and analyzing these differentially expressed genes.

## Materials and Methods

### Ethics statement

The contents of this study pertaining to the scope of medical ethics were approved by the Ethics Committee of China-Japan Union Hospital of Jilin University. Collection of samples and information were all approved by the study subjects who provided signed informed consent.

### Subjects

In total, 12 AMI patients treated at the China-Japan Union Hospital of Jilin University from June 2012 to August 2012, as well as 12 healthy individuals, were selected randomly. The diagnostic criteria of AMI were in accordance with the guidelines issued by the American Heart Association/American College of Cardiology Foundation in 2013 (O’Gara, *et al.*, 2013). A patient could be diagnosed with MI upon meeting one of the following four standards combined with the changes in typical myocardial necrotic markers (such as troponin T, or troponin I): 1. clear ischemic symptoms; 2. dynamic electrocardiograph (ECG) changes and pathological Q wave; 3. new ST-T changes in ECG, or new onset of left bundle branch block; 4. segmental wall motion disorder in imaging, or new loss of viable myocardium. The healthy individuals in the control group had no history of myocardial ischemia and had normal ECG, cardiac color Doppler, cardiac enzymes, and treadmill exercise test results. Patients with diabetes, renal insufficiency, peripheral arterial disease, or stroke were excluded.

### Microarray detection and analysis

Three AMI patients and three healthy individuals were randomly selected from all the subjects for microarray analysis: From each subject, 2 ml of peripheral blood was sampled, followed by addition of 2 ml of Trizol and storage at -80 °C before performing the microarray (Beijing Dingguo Changsheng Biotechnology Co. Ltd., Beijing, China). The microarray platform used in this test was the GPL570 HG-U133 microarray (Affymetrix, USA) with 11,800 probes, and the microarray was performed by Beijing Dingguo Changsheng Biotechnology Co. Ltd. Samples underwent first-strand and second-strand cDNA synthesis, *in vitro* transcription, biotin labeling of cRNA, and cRNA fragmentation, followed by microarray hybridization. Microarray analysis was performed using an Affymetrix GeneChip® Scanner 3000 (Affymetrix, Santa Clara, CA, USA) according to the manufacturer’s protocol. The raw data (*.CEL files) obtained from Scanner 3000 were normalized using significant analysis of microarrays (SAM), and the present or absent calls of each probe set were determined by the MAS5 method using the “affy” package in R. The differentially expressed genes between the AMI and healthy groups were identified by performing a moderated *t*-statistic in the “limma” package of R, and the raw *p* values were adjusted by the false discovery rate (FDR) method. The screened genes with differential expression were then subjected to GeneOntology (GO) and pathway analysis. The baseline information of the six subjects was processed for detailed statistics, and the main data were statistically analyzed; there were no statistical differences between the two groups with respect to age, gender, and other parameters (Table 1).

**Table 1:** Baseline information of the study subjects for microarray assay.

Parameters	AMI group (n=3)	Control group (n=3)	*P*
Age(years)	53.0 ± 13.1	53.7 ± 4.7	0.938
Gender(M/F)	2/1	3/0	1.000
HDL-C(mmol/l)	1.46 ± 0.95	1.41 ± 0.16	0.940
LDL-C(mmol/l)	2.18 ± 0.84	2.31 ± 0.74	0.846
Smoking history(yes/no)	2/1	1/2	1.000
Drinking history(yes/no)	0/3	1/2	1.000
History of Statins administration(yes/no)	0/3	0/3	1.000
Family history(yes/no)	0/3	0/3	1.000

### cDNA synthesis and real-time PCR

The remaining samples in each group were used for real-time PCR verification: 2 ml of fasting venous blood was sampled in the early morning into EDTA-anticoagulant tubes for separating lymphocytes using a lymphocyte separation liquid (Tianjin HaoYang Biological Manufacture Co., Ltd., Tianjin, China). The separated lymphocytes were then used for RNA extraction using the classical Trizol method performed as specified by the manufacturer’s instructions. The purity and concentration of RNA were evaluated by agarose gel electrophoresis and using a UV spectrophotometer; samples with a A260/280 value ranging from 1.8 to 2.0 were considered qualified samples. According to the instructions in TOYOBO ReverTra Ace kit (TOYOBO, Dalian, China), the obtained RNA was reverse-transcribed to cDNA, which was then stored at -20 °C for RT-qPCR detection. RT-qPCR was performed using the modified TIANGEN fluorescence quantitation pre- mixed reagent kit (Tiangen Biotechnology Co., Ltd., Beijing, China), and the reaction conditions were: 1 cycle at 95 °C for 15 min followed by 40 cycles of 95 °C for 10 s and 62 °C for 30 s. The reactions were performed on an Mx3000P quantitative PCR instrument (Shanghai GeneTimes Technology Inc., Shanghai, China). Certain differentially expressed genes, such as *CYP4F3/TBL1XR1/GBGT1* (up-regulated), *USP25/FDFT1/RORA* (down-regulated), and *IL13RA1* (not regulated), were randomly selected for RT-qPCR verification. *GAPDH* was used as the internal (endogenous) control, and the 2^-ΔΔCt^ method was applied to determine the difference in relative expression. The primer sequences used for each gene are shown in Table 2.

**Table 2:** Primer sequences for fluorescence quantitation.

Gene		Primer sequence(5'−>3')
CYP4F3	F	ATTGGTTCTTGGGTCACCTG
	R	GATGTAGGTGGGGTGGAAGA
TBL1XR1	F	CACCCGCTGCATTGATTTCTA
	R	TACGGCATCTATCAGGGACAG
GBGT1	F	TGGGTGTATCTTGAGAACTGGC
	R	GTACTGTGACCATACCACGGG
USP25	F	GATGAAAGGTGTCACAACATAATGAAA
	R	CCACTCCTCATATTCCTCCAAGTTT
FDFT1	F	ACTTCCCAACGATCTCCCTTG
	R	CCCATTCTCCGGCAAATGTC
RORA	F	ACTCCTGTCCTCGTCAGAAGA
	R	CATCCCTACGGCAAGGCATTT
IL13RA1	F	TCCAATTCCTGATCCTGGCAAGATT
	R	TTCTATCAGCACTACAGAGTCGGTT
GAPDH	F	CTCCTGGAAGAT GGTGATGG
	R	ACGGATTTGGTC GTATTGGGCG

### Statistical analysis

SPSS17.0 was used for statistical analysis; the quantitative data are expressed as mean ± standard deviation, and the intergroup difference was analyzed by the *t*-test; qualitative data are expressed using “frequency,” and intergroup difference was determined by Fisher’s exact test. *P* < 0.05 indicated statistical significance.

## Results

### Baseline information

The baseline data for the patients whose samples were used in RT-q PCR verification are shown in Table 3. The two groups showed no statistically significant difference with respect to age, gender, or blood lipid content.

**Table 3:** Baseline information of the study subjects for real-time fluorescence quantitative PCR verification.

Parameters	AMI group (n=9)	Control group (n=9)	*P*
Age (years)	58.812.8	58.3 ± 16	0.943
Gender (M/F)	7/2	6/3	1.000
HDL-C (mmol/l)	1.65 ± 0.74	1.26 ± 0.19	0.145
LDL-C (mmol/l)	2.38 ± 0.64	2.36 ± 0.37	0.936
Smoking history (yes/no)	2/7	2/7	1.000
Drinking history (yes/no)	2/7	3/6	1.000
History of Statins administration (yes/no)	0/9	1/8	1.000
Family history (yes/no)	1/8	0/9	1.000

### Results of microarray analysis

**Figure 1:**
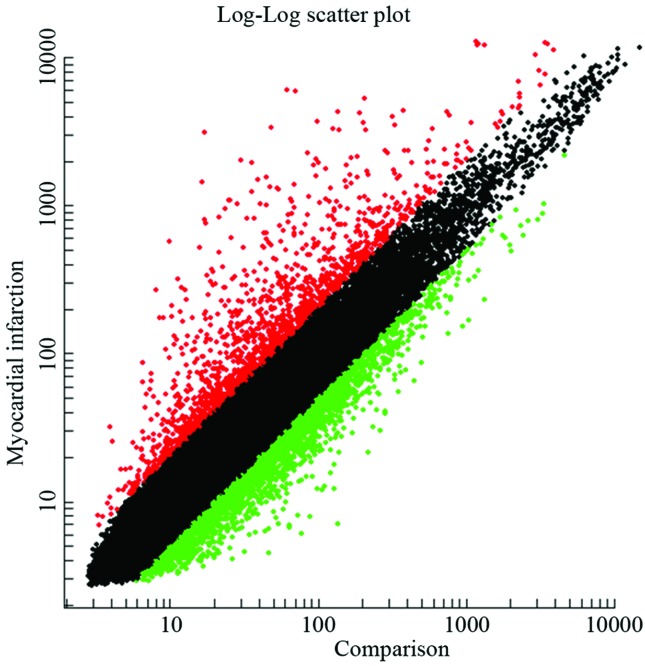
Scatterplot of differentially expressed genes between AMI and healthy people.

The results of the microarray in this study were analyzed using SAM with *P* ≤ 0.05 and |logFC| > 1 as the screening criteria. In total, 559 RNA fragments showed differential expression between the AMI and control groups, of which 271 genes were down- regulated and 288 were up-regulated (Figure 1). Red dots represent the up-regulated genes, green dots represent the down-regulated genes, and black dots represent the unaltered genes. The top differentially expressed genes are shown in Table 4. The details of the gene chips have been uploaded to the Gene Expression Omnibus (GEO) database for consultation: GEO series accession number GSE97320 (https://www.ncbi.nlm.nih.gov/geo/query/acc.cgi?acc=GSE97320).

**Table 4:** The top differentially expressed genes.

Gene Symbol	logFC	*P*
FRG1JP	-2.40024	0.00023
LOC202181	-1.83279	0.001522
PTGS2	2.901423	0.001881
TNFRSF10C	2.295785	0.002082
LIN7A	1.664351	0.003145
KRT23	3.612449	0.003452
PRKCI	-1.56355	0.003518
ABCC4	1.822611	0.003814
DZIP3	-1.985	0.004079
VNN3	1.724204	0.004178
LOC105377200	-1.85184	0.004275
NFE4	2.009371	0.004284
ENOSF1	-1.29599	0.004697
SLAIN2	-1.43069	0.004855
GBGT1	1.642487	0.004933
MMP25	2.366192	0.005301
THBD	2.157065	0.005327
CXCL5	2.92028	0.005388
G0S2	2.437448	0.005758
NMT2	-2.18653	0.00612
SMIM14	-1.2562	0.006733
GVINP1	-1.23053	0.00683
ADM	2.490298	0.007169
PI3	3.355852	0.007782
MBOAT7	2.153989	0.007905
LINC00152	1.528425	0.007979
C4A	1.890805	0.007996
FGFR1OP2	-1.31358	0.008
FPR1	1.912927	0.008116
SLPI	2.47279	0.008202

These differentially expressed genes were then subjected to GO analysis, and the results revealed that these genes belong to 128 categories of cellular localization, are involved in 521 biological processes, and are suggested to have 151 molecular functions (details are shown in supplementary material Tables S1-S3).

The Kyoto Encyclopedia of Genes and Genomes (KEGG) pathway analysis of the559 differentially expressed genes revealed that these genes participate in 107 gene pathways, and the primary pathways, as shown in Table S4.

### RT-qPCR analysis

In this study, the dissolution curves and amplification curves of the candidate genes and reference genes met the quantitative requirements.

**Figure 2:**
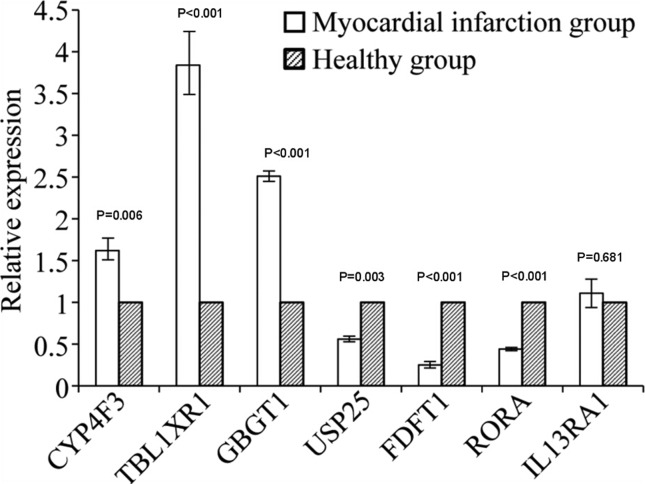
Verification result: Relative expression of the candidate genes analyzed by RT-qPCR.

The RT-qPCR results showed that transcripts of the *CYP4F3/ TBL1XR1/GBGT1* genes in the peripheral blood of the AMI group were up-regulated compared to that in the control group, whereas *USP25/RORA/FDFT1* were down-regulated compared to the control group; *IL13RA1* expression showed no significant difference between the two groups(Table 5 and Figure 2). These results showed the same trend as the results of the microarray, indicating that the microarray was accurate and could be used for screening differentially expressed genes.

**Table 5:** RT-PCR results of the candidate genes.

Gene	ΔCq		ΔΔCq	2^−ΔΔCq^	*P*
	AMI group	Control group			
CYP4F3	12.3 ± 0.11	12.98 ± 0.22	-0.68 ± 0.19	1.62 ± 0.27	0.006
TBL1XR1	2.47 ± 0.36	4.38 ± 0.07	-1.91 ± 0.29	3.84 ± 0.78	< 0.001
GBGT1	8.45 ± 0.20	9.78 ± 0.24	-1.32 ± 0.07	2.51 ± 0.13	< 0.001
USP25	7.07 ± 0.04	6.24 ± 0.17	0.83 ± 0.15	0.56 ± 0.07	0.003
FDFT1	5,24 ± 0.35	3.16 ± 0.73	2.08 ± 0.47	0.250.08	< 0.001
RORA	6.49 ± 0.48	5.29 ± 0.39	1.21 ± 0.14	0.44 ± 0.04	< 0.001
IL13RA1	5.97 ± 0.27	6.07 ± 0.27	-0.10 ± 0.27	1.11 ± 0.35	0.681

Cq: quantification cycle; ΔCq =Cq of the target gene - Cq of the endogenous control gene; ΔΔCq=ΔCq of the AMI group -ΔCq of the control group; 2^−ΔΔCq^ represents relative expression.

## Discussion

This study analyzed the differentially expressed genes in peripheral blood of patients with AMI and found 559 differentially expressed genes in the above patients, among which 288 were up-regulated and 271 were down-regulated.

Among the target genes, *CYP4F3* encodes a member of the large cytochrome P450 family, belonging to the CYP4F subfamily. P450 enzymes are wide-spectrum biological catalysts in nature and can act on a variety of substances. During the reperfusion phase, injection of nonspecific inhibitors of CYP, such as chloramphenicol, cimetidine, and sulfaphenazole (selective inhibitor of CYP2C9), could significantly reduce ROS generation in the rat heart and reduce the infarct size (Hunter *et al.*, 2005). Furthermore, the myocardial protective effects of the medication during the reperfusion phase showed that medication to patients with AMI after ischemia might still be effective, and this finding is of important clinical significance (Nithipatikom *et al.*, 2004). Moreover, 20-HETE, a metabolite of CYP4F3, is a strong contraction agent acting on small arterioles. By inhibiting the KCa channel, activating the L-type Ca^2+^ channel, and activating PKC, it could increase the intracellular Ca2+ content and cause shrinkage in small arteries. The injury might be associated with blockage of the heart sarcKATP pathway. In this study, CYP4F3 was upregulated in the AMI group, suggesting that CYP4F3 might participate in the myocardial injury and repair processes of AMI.

USP25 is a member of the USPS family and participates in all processes of tumor occurrence. It is known that USP25 is involved in the metastasis of non-small cell lung cancer cells, which is promoted by inducing *miR-200C* (Li *et al.*, 2014). Studies also showed that USP25 could negatively regulate the IL17-mediated inflammatory response (Zhong *et al.*, 2012). In this study, USP25 in the AMI group was down-regulated compared to the control group. AMI is associated with inflammation, but the detailed mechanisms by which USP25 affects the occurrence of AMI still need to be explored.

The protein encoded by the *RORA* gene is a member of the family of NR1 subunit hormone receptors and plays an important role in regulating the metabolism of lipids and glucose, as well as insulin expression (Vu-Dac *et al.*, 1997). The *RORA* gene is considered a predisposing gene for diabetes in Mexican Americans (Hayes *et al.*, 2007). Furthermore, mutation of an individual base in this gene is positively correlated with incidence of diabetes in the Chinese population (Zhang *et al.*, 2016). The protein encoded by the *FDFT1* gene is the first enzyme in cholesterol biosynthesis, and studies have found that this gene can affect blood lipids, blood sugar, and inflammation, thus participating in obesity-related coronary heart disease, diabetes, and coronary artery calcification (Ding *et al.*, 2015).

Other than the target genes, genes showing high differential expression included *PTGS2,* encoding a cyclooxygenase that is a key enzyme in prostaglandin biosynthesis and is thought to be involved in the occurrence of myocardial infarction, hypertension, and diabetes. Studies have shown that polymorphism in the *PTGS2* gene reduces the risk of myocardial infarction and stroke by affecting COX-2 activity and reducing the formation of atherosclerotic plaques (Cipollone *et al.*, 2004). Relevant experiments have shown that the rs20417 mutant of the *PTGS2* gene can significantly reduce the risk of cardiovascular events (Ross *et al.*, 2014). Animal experiments have demonstrated that inhibiting *PTGS2* expression can increase susceptibility to salt-sensitive hypertension (Zhang MZ *et al.*, 2015). The protein encoded by the *PRKCI* gene is one of the members of the protein kinase C family, which is known to affect glucose degradation by participating in insulin-mediated glucose transport (Bandyopadhyay *et al.*, 2004). In the platelet activation pathway, the activated PRKCI protein activates the Ras-associated protein, RAP-1a, thereby indirectly promoting platelet aggregation. Among the screened genes, a large part has been found to be associated with tumor occurrence or participating in biological processes such as RNA degradation; yet not all genes were found to be related to the formation of myocardial infarction, and the roles of some genes are not yet clear. The impact of these differentially expressed genes and their expression changes on the formation of myocardial infarction still needs further verification.

Compared with genes, pathways may play a more important role in the onset of AMI. The KEGG pathway analysis showed that the 559 differentially expressed genes participated in 107 pathways, including the systemic lupus erythematosus pathway, apoptosis, mitogen-activated protein kinase (MAPK) signaling, and insulin signaling. Systemic lupus erythematosus (SLE) is an autoimmune disease involving multiple systems and multiple organs, and the expression of a variety of autoantibodies. The autoantibodies deposited in renal glomeruli and autoantigen immune complexes mediate systemic inflammatory responses by activating complement proteins or neutrophils and macrophages via FcγR. SLE was found to have the same risk factors as MI, including hypertension, hyperlipidemia, smoking, or diabetes (Petri *et al.*, 1992). Patients with SLE were also at higher risk of cardiovascular diseases (Tazi Mezalek *et al.*, 2014; Schuett *et al.*, 2015). A study in Taiwan revealed that the risk ratio of AMI combined with SLE was 5.11, which was 6.28 in females, and MI patients with SLE exhibited a higher mortality rate (Lin *et al.*, 2014). The existence of SLE could cause blood vessel inflammation, thus inducing vascular remodeling and platelet aggregation, as well as atherosclerosis (Quinlan *et al.*, 2016). The pathway analysis revealed the most differentially expressed genes in SLE (17 genes), which is consistent with related reports indicating that these differentially expressed genes lead to disorders of related inflammation adjustment due to expression changes, thus affecting the occurrence of MI.

MAPK is a group of serine/threonine protein kinases that can be activated by different extracellular stimuli, such as cytokines, neurotransmitters, hormones, cell stress, or cell adhesion. The MAPK pathway included MAP kinase kinase kinase (MKKK), MAP kinase kinase (MKK), and MAPK; these three kinases can be activated consecutively and co-regulate a variety of important cellular physiological/ pathological processes, such as cell growth, differentiation, adaptation to environmental stress, and inflammation. P38-MAPK is an important apoptotic mediator, and after ischemia, P38 can be rapidly activated through phosphorylation, and its concentration could be increased by reperfusion (Sanchez *et al.*, 2012). In addition, p38-MAPK is involved in a variety of inflammatory reactions, including the reaction process of myocardial injury (Gao *et al.*, 2015; Guo *et al.*, 2015). In this study, eight differentially expressed genes were involved in the normal transduction of this signaling pathway, and abnormal expression of these genes was involved in the occurrence of MI and the formation of reperfusion injury.

The toll-like receptor signaling pathway includes receptors using different modes that can detect bacteria, viruses, fungi, and parasites, using pathogen-associated molecular modes. Each receptor binds to a specific ligand, initiates an innate immune response towards a particular pathogen, and then activates the acquired immune response. Studies have shown that TLR4-mediated signals may induce myocardial dysfunction during myocardial ischemia/reperfusion (Li *et al.*, 2015). Toll-like receptor 9 plays an important role in the development of stress-induced inflammation and heart failure (Omiya *et al.*, 2016).

It is well known that diabetes is an independent risk factor for MI. The insulin signaling pathway can regulate the glucose content of the body by over-regulating the degradation and transformation of glucose, and indirectly influence lipid synthesis, thus affecting the occurrence of myocardial infarction. Glucose is the main energy substrate supporting myocardial contraction, which can increase myocardial contraction, and insulin can regulate this by regulating the blood sugar balance. Moreover, insulin can directly act on the myocardium by mediating the Akt signaling pathway, stimulating the production of vascular endothelial growth factors and vessels, inhibiting apoptosis, promoting cell survival, and ultimately improving myocardial microcirculation and coronary artery resistance by increasing the synthesis of ribosomes and proteins, thereby increasing myocardial blood flow (Iliadis *et al.*, 2011). It was found that strictly controlling blood glucose after myocardial infarction can reduce the senescent myocyte precursor cells, thus increasing the possibility of recovery of the ischemic myocardium (Marfella *et al.*, 2012).

Owing to funding constraints, the gene chips in this study were limited, and the number and size of samples used for verification was relatively small. However, the verification results were consistent with the microarray results, indicating that the microarray results were relatively accurate, and could be used for analyzing AMI-related pathways and screening the target genes. This study used clinically obtained venous blood as samples, which may facilitate clinical diagnosis and treatment using the discovered target genes in the future.

## Conclusions

Gene expression differences were observed in the peripheral blood of patients with AMI and healthy individuals. The microarray revealed 559 differentially expressed genes in the peripheral blood of Northeast Chinese Han patients with AMI. RT-qPCR verified that the results of the microarray were relatively accurate and could be used for screening differentially expressed genes. Abnormal regulation of SLE and MAPK metabolic pathways might have a significant impact on the occurrence of MI. *CYP4F3/TBL1XR1/GBGT1/USP25/FDFT1/RORA* expression could possibly be used as markers for the diagnosis of AMI, and hopefully, suggest new methods when being used as clinical targets for the treatment of AMI patients.

## Supplementary material

The following online material is available for this article:

Table S1 - GO analysis – Cellular localization.

Table S2 - GO analysis – Biological process.

Table S3 - GO analysis – Molecular function.

Table S4 - Main KEGG pathways.
