# Supplementary material for: Differential gene expression profiles in peripheral blood in Northeast Chinese Han people with acute myocardial infarction
Source: Genet Mol Biol. 2018 Jan-Mar;41(1):59–66. doi: 10.1590/1678-4685-GMB-2017-0075 (PMC5901496; doi:10.1590/1678-4685-GMB-2017-0075)
Supplement: Supplementary file 1 [file 1415-4757-GMB-41-01-2017-0075-s001.pdf]

Supplementary Material to "Differential gene expression profiles in peripheral blood in Northeast Chinese Han people with acute myocardial infarction"

Table S1 - GO analysis – Cellular localization.

GO Term	Count	p-Value	q-Value
Nucleus	117	2.45E-120	1.47E-118
Cytoplasm	104	6.56E-87	1.31E-85
Integral to membrane	63	7.52E-46	7.52E-45
Plasma membrane	54	1.61E-44	1.38E-43
Membrane	67	1.61E-42	1.21E-41
Cytosol	25	3.83E-27	1.77E-26
Golgi apparatus	21	1.49E-22	5.25E-22
Integral to plasma membrane	22	8.10E-21	2.70E-20
Extracellular region	26	4.79E-20	1.51E-19
Extracellular space	15	7.38E-17	2.01E-16
Intracellular	44	1.95E-14	4.67E-14
Mitochondrion	16	3.18E-14	7.33E-14
Endoplasmic reticulum	15	6.81E-14	1.46E-13
Hemoglobin complex	5	8.90E-13	1.67E-12
Nucleoplasm	13	1.04E-12	1.89E-12

GO Term	Count	p-Value	q-Value
Cytoskeleton	15	7.97E-12	1.26E-11
Golgi membrane	10	1.20E-11	1.85E-11
Nucleolus	9	5.51E-09	6.01E-09
Anchored to membrane	6	6.15E-09	6.59E-09
Endoplasmic reticulum membrane	9	8.23E-09	8.66E-09
Chromosome	7	1.50E-07	1.23E-07
Membrane fraction	8	3.97E-07	3.05E-07
Nucleosome	4	7.15E-07	5.43E-07
Microsome	5	1.37E-06	1.02E-06
Centrosome	5	2.37E-06	1.64E-06
Soluble fraction	5	2.94E-06	1.98E-06
Cell junction	6	1.17E-05	7.08E-06
Basolateral plasma membrane	4	1.94E-05	1.14E-05
Nuclear speck	3	1.15E-04	5.68E-05
Microtubule	4	1.18E-04	5.74E-05
