# Supplementary material for: Differential gene expression profiles in peripheral blood in Northeast Chinese Han people with acute myocardial infarction
Source: Genet Mol Biol. 2018 Jan-Mar;41(1):59–66. doi: 10.1590/1678-4685-GMB-2017-0075 (PMC5901496; doi:10.1590/1678-4685-GMB-2017-0075)
Supplement: Supplementary file 2 [file 1415-4757-GMB-41-01-2017-0075-s003.pdf]

Supplementary Material to "Differential gene expression profiles in peripheral blood in Northeast Chinese Han people with acute myocardial infarction"

Table S3 - GO analysis—Molecular function.

GO Term	Count	p-Value	q-Value
Protein binding	115	1.85E-99	5.55E-98
Zinc ion binding	54	1.12E-54	1.69E-53
Metal ion binding	64	4.18E-54	5.02E-53
Nucleotide binding	35	3.46E-31	2.07E-30
DNA binding	35	2.85E-29	1.56E-28
Transferase activity	27	1.19E-23	4.75E-23
ATP binding	19	4.98E-16	1.30E-15
Ligase activity	12	9.48E-15	2.37E-14
RNA binding	14	4.07E-14	9.06E-14
Calcium ion binding	15	9.18E-14	1.90E-13
Receptor activity	18	4.73E-13	9.15E-13
Magnesium ion binding	11	1.74E-12	2.99E-12
Transcription factor activity	14	2.09E-12	3.48E-12
GTP binding	10	7.26E-12	1.18E-11

GO Term	Count	p-Value	q-Value
Oxidoreductase activity	11	1.54E-10	2.20E-10
Manganese ion binding	7	3.01E-10	4.11E-10
Peptidase activity	10	6.68E-10	8.53E-10
Oxygen transporter activity	4	7.88E-10	9.66E-10
Protein serine/threonine kinase activity	9	8.05E-10	9.66E-10
Hydrolase activity	16	2.59E-09	3.04E-09
Heme binding	6	3.80E-09	4.38E-09
Ubiquitin-protein ligase activity	6	5.34E-09	5.94E-09
G-protein coupled receptor activity	10	3.17E-08	2.97E-08
Iron ion binding	6	4.15E-08	3.72E-08
Transcription coactivator activity	6	4.43E-08	3.86E-08
Oxygen binding	4	4.43E-08	3.86E-08
GTPase activity	6	6.09E-08	5.15E-08
Chemokine activity	4	1.91E-07	1.53E-07
Transcription corepressor activity	5	2.12E-07	1.68E-07
Helicase activity	5	3.78E-07	2.94E-07
