# Supplementary material for: Differential gene expression profiles in peripheral blood in Northeast Chinese Han people with acute myocardial infarction
Source: Genet Mol Biol. 2018 Jan-Mar;41(1):59–66. doi: 10.1590/1678-4685-GMB-2017-0075 (PMC5901496; doi:10.1590/1678-4685-GMB-2017-0075)
Supplement: Supplementary file 3 [file 1415-4757-GMB-41-01-2017-0075-s004.pdf]

Supplementary Material to “Differential gene expression profiles in peripheral blood in Northeast Chinese Han people with acute myocardial infarction”

Table S4 - Main KEGG pathways.

Pathway	Count	p-Value	q-Value
Systemic lupus erythematosus	17	9.30E-18	2.33E-16
Toll-like receptor signaling pathway	10	3.65E-10	2.10E-09
Epithelial cell signaling in Helicobacter pylori infection	7	1.47E-07	5.85E-07
Cytokine-cytokine receptor interaction	10	2.75E-06	7.77E-06
Bladder cancer	4	9.30E-05	1.29E-04
Insulin signaling pathway	6	1.39E-04	1.74E-04
Apoptosis	5	1.54E-04	1.89E-04
MAPK signaling pathway	8	1.83E-04	2.14E-04
Pathogenic Escherichia coli infection - EHEC	4	2.68E-04	2.85E-04
Pathogenic Escherichia coli infection - EPEC	4	2.68E-04	2.85E-04
Amyotrophic lateral sclerosis (ALS)	4	2.87E-04	2.92E-04
Cell cycle	5	5.91E-04	5.40E-04
Leukocyte transendothelial migration	5	6.37E-04	5.69E-04
Complement and coagulation cascades	4	6.38E-04	5.69E-04

Pathway	Count	p-Value	q-Value
Fc epsilon RI signaling pathway	4	0.00106075	8.94E-04
Ubiquitin mediated proteolysis	5	0.001187375	9.68E-04
Small cell lung cancer	4	0.001453884	0.001135847
TGF-beta signaling pathway	4	0.001517287	0.001161189
Porphyrin and chlorophyll metabolism	3	0.001607304	0.001217655
Aminophosphonate metabolism	2	0.001680799	0.001235882
N-Glycan biosynthesis	3	0.001845416	0.001318154
Glycosphingolipid biosynthesis - ganglioseries	2	0.003161465	0.001959586
GnRH signaling pathway	4	0.003223114	0.001981423
Neuroactive ligand-receptor interaction	6	0.003449435	0.002053235
Tight junction	4	0.00750531	0.003669751
Renal cell carcinoma	3	0.007614698	0.003684531
Glutamate metabolism	2	0.008037671	0.003803314
Pancreatic cancer	3	0.008218533	0.003852437
Chronic myeloid leukemia	3	0.008849768	0.004071979
VEGF signaling pathway	3	0.00917574	0.004183468
