# Supplementary material for: Differential gene expression profiles in peripheral blood in Northeast Chinese Han people with acute myocardial infarction
Source: Genet Mol Biol. 2018 Jan-Mar;41(1):59–66. doi: 10.1590/1678-4685-GMB-2017-0075 (PMC5901496; doi:10.1590/1678-4685-GMB-2017-0075)
Supplement: Table S2 [file 1415-4757-GMB-41-01-2017-0075-s002.pdf]

Supplementary Material to “Differential gene expression profiles in peripheral blood in Northeast Chinese Han people with acute myocardial infarction”

Table S2 - GO analysis — Biological process.

GO Term	Count	p-Value	q-Value
Regulation of transcription, DNA-dependent	41	1.42E-36	9.44E-36
Transcription	33	2.12E-25	9.07E-25
Signal transduction	29	4.59E-17	1.31E-16
Inflammatory response	19	1.28E-27	6.38E-27
Immune response	19	3.06E-19	9.18E-19
Chemotaxis	14	4.70E-23	1.76E-22
Oxidation reduction	13	1.49E-13	2.98E-13
Cell cycle	12	1.91E-10	2.67E-10
Development	12	1.14E-05	7.00E-06
Ion transport	11	4.24E-10	5.65E-10
G-protein coupled receptor protein signaling pathway	11	2.35E-08	2.27E-08
Interspecies interaction between organisms	10	1.71E-12	2.99E-12
Modification-dependent protein catabolism	10	5.27E-10	6.88E-10
Apoptosis	10	3.61E-08	3.33E-08

GO Term	Count	p-Value	q-Value
Response to DNA damage stimulus	9	1.04E-10	1.52E-10
Cell-cell signaling	9	2.21E-08	2.21E-08
Protein amino acid phosphorylation	9	5.65E-08	4.84E-08
Protein modification	9	2.50E-05	1.43E-05
Transport	9	0.002129061	4.53E-04
Cell motility	8	3.92E-08	3.56E-08
Carbohydrate metabolism	8	1.11E-07	9.25E-08
Cell adhesion	8	1.76E-06	1.26E-06
Cell surface receptor linked signal transduction	8	7.01E-04	2.68E-04
Ubiquitin-dependent protein catabolism	7	4.76E-09	5.38E-09
Negative regulation of cell proliferation	7	2.32E-08	2.27E-08
RNA splicing	7	2.38E-08	2.27E-08
Vesicle-mediated transport	7	1.66E-06	1.22E-06
Cell proliferation	7	4.43E-05	2.39E-05
Mitosis	6	1.73E-07	1.40E-07
Regulation of transcription from RNA polymerase II promoter	6	2.79E-05	1.58E-05
